# AMPK-induced activation of Akt by AICAR is mediated by IGF-1R dependent and independent mechanisms in acute lymphoblastic leukemia

**DOI:** 10.1186/1750-2187-5-15

**Published:** 2010-09-23

**Authors:** Gilles M Leclerc, Guy J Leclerc, Guilian Fu, Julio C Barredo

**Affiliations:** 1 Department of Pediatric Hematology-Oncology, University of Miami Miller School of Medicine, 1601 N.W. 12th Avenue, Miami, FL 33136 USA; 2 Department of Biochemistry and Molecular Biology, University of Miami Miller School of Medicine, 1011 N.W. 15th Street, Miami, FL 33101 USA; 3 Sylvester Comprehensive Cancer Center, University of Miami Hospital and Clinics, 1475 N.W. 12th Avenue, Miami, FL 33136 USA

## Abstract

**Background:**

Children with Acute Lymphoblastic Leukemia (ALL) diagnosed with resistant phenotypes and those who relapse have a dismal prognosis for cure. In search for novel treatment strategies, we identified the AMP activated protein kinase (AMPK) as a potential drug target based on its effects on cell growth and survival. We have shown previously that AICAR-induced AMPK activation also induced a compensatory survival mechanism via PI3K/Akt signaling.

**Results:**

In the present study, we further investigated the downstream signaling induced by AMPK activation in ALL cells. We found that AICAR-induced AMPK activation resulted in up-regulation of P-Akt (Ser473 and Thr308) and decrease of P-mTOR (Ser2448) expression and downstream signaling. We determined that activation of P-Akt (Thr308) was mediated by AMPK-induced IGF-1R activation via phosphorylation of the insulin receptor substrate-1 (IRS-1) at Ser794. Inhibition of IGF-1R signaling using the tyrosine kinase inhibitor HNMPA(AM)_3 _resulted in significant decrease in P-IRS-1 (Ser794) and P-Akt (Thr308). Co-treatment of AICAR plus HNMPA(AM)_3 _prevented AMPK-induced up-regulation of P-Akt (Thr308) but did not alter the activation of P-Akt (Ser473). Inhibition of AMPK using compound-C resulted in decreased P-Akt expression at both residues, suggesting a central role for AMPK in Akt activation. In addition, inhibition of IGF-1R signaling in ALL cells resulted in cell growth arrest and apoptosis. Additional Western blots revealed that P-IGF-1R (Tyr1131) and P-IRS-1 (Ser794) levels were higher in NALM6 (Bp-ALL) than CEM (T-ALL), and found differences in IGF-1R signaling within Bp-ALL cell line models NALM6, REH (TEL-AML1, [t(12;21)]), and SupB15 (BCR-ABL, [t(9;22)]). In these models, higher sensitivity to IGF-1R inhibitors correlated with increased levels of IGF-1R expression. Combined therapy simultaneously targeting IGF-1R, AMPK, Akt, and mTOR pathways resulted in synergistic growth inhibition and cell death.

**Conclusions:**

Our study demonstrates that AMPK activates Akt through IGF-1R dependent and independent mechanisms. Co-targeting IGF-1R and related downstream metabolic and oncogenic signaling pathways represent a potential strategy for future translation into novel ALL therapies.

## Background

Acute Lymphoblastic Leukemia (ALL) is the most common hematological malignancy affecting children and adolescents, and remains the leading cause of cancer-related mortality in this age group [1]. ALL is a heterogeneous disease with distinct phenotypes segregated by the presence of non-random translocations and genomic deletions and amplifications [2]. Despite significant progress in the treatment of ALL, a large number of children continue to relapse and for them, outcome remains poor. In addition, adults are generally diagnosed with resistant phenotypes of ALL and continue to respond poorly to existing treatment regimens. Therefore, novel therapies need to be developed. Recently, our laboratory identified AMP activated protein kinase (AMPK) as a potential target for ALL therapy due to its effects on cell growth and its signaling crosstalk with critical metabolic and oncogenic pathways [3]. Treatment with the AMPK activator 5-aminoimidazole-4-carboxamide-1-β-D-ribofuranoside (AICAR) induced apoptotic cell death in ALL cells mediated by AMPK, mTOR, P27, P53, and p38-MAPK [3]. In addition, AICAR significantly increased P-Akt (Ser473) following AMPK activation and mTOR down-regulation, which was viewed as a compensatory survival mechanism. Akt (protein kinase B) is involved in critical survival pathways, and inhibits apoptosis via phosphorylation of the pro-apoptotic protein BAD at Ser136, which prevents its inhibitory association with the anti-apoptotic Bcl-2 protein [4-6]. Akt is activated by phosphorylation of two key residues: Thr308 within the T-loop of its catalytic domain, and Ser473 located in the hydrophobic region of its C-terminal domain [7,8]. Phosphorylation of both residues is essential for maximal activity [8] and was found to be regulated by independent mechanisms [9]. Phosphorylation of Akt at Ser473 involves rictor, a member of the TORC2 complex known to modulate the activity of mTOR [7,10-12], while phosphorylation of Thr308 is mediated by PDK1 and PIP3 following phosphorylation of PIP2 by PI3K [13,14]. The latter mechanism is responsible for the described feedback loop inhibition of Akt phosphorylation mediated by mTOR-dependent phosphorylation of IRS-1 at Ser312, the immediate downstream effector protein of the insulin-like growth factor-1 receptor (IGF-1R) [15,16]. Phosphorylation of IRS-1 (Ser312) by P-mTOR promotes conformational changes and subsequent detachment from the receptor and degradation [17], and inhibits potentiation of Akt by IGF-1R/IRS-1 signaling [18]. Conversely, inhibition of mTOR results in IRS-1 activation and increased phosphorylation of Akt at Thr308 [19].

IGF-1R is one of four transmembrane receptors (IGF-1R, IGF-IIR, IR, and hybrids receptors of IGF and IR) that compose the IGF-1R signaling system in addition to the three circulating ligands (IGF-I, IGF-II, and insulin) and multiple regulatory IGF-binding proteins (IGFBP-1 to -6) [20-23]. IGF-1R is ubiquitously expressed in human cancer cells compared to normal tissues [24]. Elevated plasma concentrations of IGF-1, IGFBP-2, and IGFBP-3 have been linked to more aggressive phenotypes in breast, colon, prostate, lung cancer, and ALL [25,26]. IGF-1R exerts its action through activation of downstream signaling cascades that regulate metabolic and oncogenic pathways important for cellular growth [27]. IGF-1R signaling has been linked to the regulation of normal and malignant hematopoietic cells. Significant differences in the expression of the IGF-1 system components IGF-II, IGFBP-2, IGFBP-4 and IGFBP-5 have been found between B-lineage and T-lineage ALL [28-30]. Taken together, this suggests that activation of IGF-1R signaling and its downstream pathways may confer ALL cells a survival advantage by influencing growth and metabolic adaptations aimed at supporting accelerated growth. Therefore, to delineate the mechanism responsible for ALL cell survival regulated by AMPK and IGF-1R and to understand the role of IGF-1R in this process, we investigated the relationship between AMPK and the cell proliferation and survival pathways downstream of IGF-1R/IRS-1. As a result, we uncovered potential combination therapies that simultaneously target key factors within these signaling cascades.

## Results

### AICAR-induced AMPK activation promotes phosphorylation of IRS-1 at Ser794

**Figure 1:**
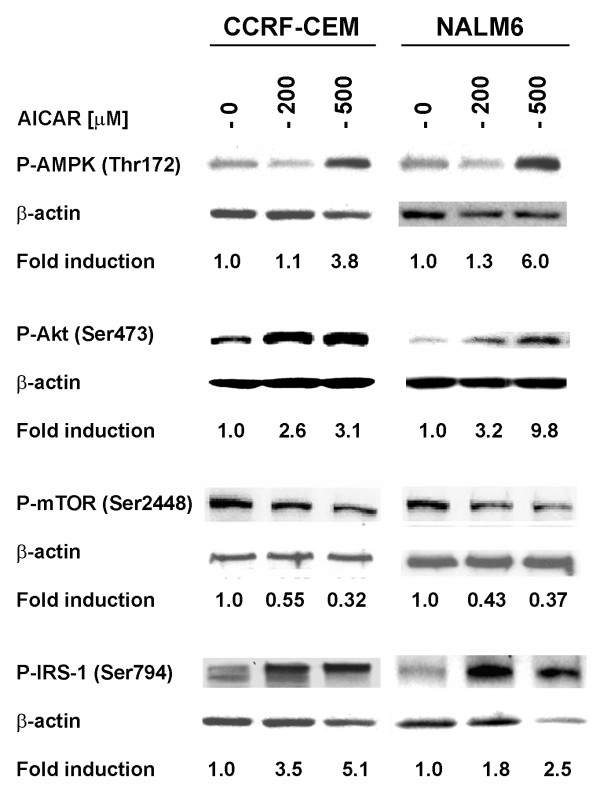
**Activation of AMPK induces phosphorylation of IRS-1 at Ser794 and down-regulation of mTOR (Ser2448) in ALL cell lines**. Western blot analysis of the expression of AMPK (Thr172), Akt (Ser473), mTOR (Ser2448) and IRS-1 (Ser794) in CCRF-CEM (T-ALL) and NALM6 (Bp-ALL) cells treated with AICAR (200 and 500 μM) and incubated for 24 h at 37°C. The density value of each band was normalized to β-actin level and expressed relative to control (shown as fold induction).

Recently, we reported that treatment of ALL cell lines with AICAR induced growth inhibition and apoptosis, and resulted in increased expression of P-Akt (Ser473) [3]. Phosphorylation of Akt, especially at the Ser473 residue, has been shown to be regulated by the mTOR/TORC2 complex [7,10-12], whereas phosphorylation of Akt at Thr308 was shown to be regulated by mTOR but through a feedback loop inhibition mechanism targeting IRS-1 [15-17]. To investigate the role of AMPK and mTOR in this process, we examined the levels of P-mTOR (Ser2448) and P-IRS-1 (Ser794, a residue known to be phosphorylated by AMPK) [31] in CCRF-CEM (T-ALL) and NALM6 (Bp-ALL) cells treated with AICAR. As expected, levels of P-AMPK and P-Akt (Ser473) were increased following treatment with AICAR (200 and 500 μM), while expression of P-mTOR (Ser2448) was significantly decreased (*p *< 0.001, for control *vs*. AICAR treated cells) (Fig. 1). Concomitantly, expression of P-IRS-1 (Ser794) was significantly increased in a dose dependent manner (*p *< 0.05, for control *vs*. AICAR treated cells). These changes in phosphorylated protein expression directly correlated with level of P-AMPK (Thr172), and inversely correlated with the degree of P-mTOR down-regulation (Fig. 1). These data indicate that the compensatory increase in P-Akt expression seen in AICAR-treated ALL cells results from both activation of IRS-1 by AMPK, and inhibition of the mTOR mediated feedback loop inhibition of IRS-1 activity. Nevertheless, as previously demonstrated, this compensatory up-regulation of P-Akt was unable to rescue ALL cells from apoptotic death following AICAR-induced AMPK activation [3].

### AICAR-induced phosphorylation of Akt at Ser473 is independent of IGF-1R/IRS-1 signaling in ALL but requires AMPK activation

**Figure 2:**
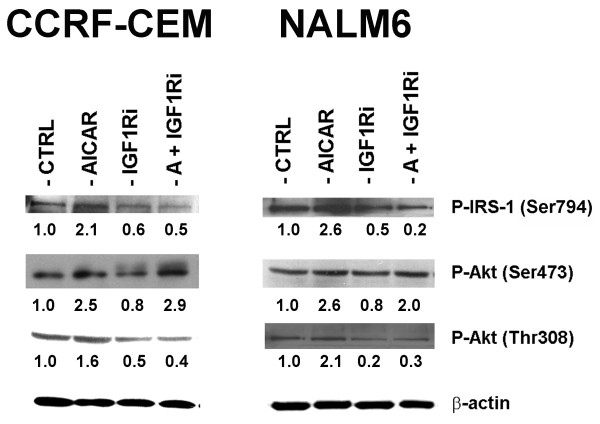
**AICAR-induced AMPK activation phosphorylates Akt at Ser473 in a mechanism independent of IGF-1R/IRS-1 signaling in ALL cell lines**. Western blot analysis of P-IRS-1 (Ser794), P-Akt (Ser473), and P-Akt (Thr308) in CCRF-CEM and NALM6 cells treated with 0.1% DMSO (CTRL), AICAR (200 μM), the IGF-1R tyrosine kinase inhibitor HNMPA(AM)_3 _(IGF1Ri, 10 μM), or both agents (A + IGF1Ri) and incubated for 24 h at 37°C. The levels of P-IRS-1 and P-Akt were normalized to β-actin (loading control) and expressed relative to control (shown as fold induction).

To characterize the extent to which the increase in P-Akt expression was dependent on IGF-1R/IRS-1, we used the specific tyrosine kinase inhibitor HNMPA(AM)_3 _(IGF1Ri) to inhibit IGF-1R/IRS-1 signaling, and examined its effects on P-IRS-1 (Ser794) and P-Akt (Ser473 and Thr308) expression in AICAR-treated CCRF-CEM and NALM6 cells using Western immunoblotting. As shown in Fig. 2, treatment with AICAR (200 μM) alone for 24 h increased the expression of P-IRS-1 (Ser794) and P-Akt at Ser473 and Thr308 by over two fold, whereas treatment with HNMPA(AM)_3 _alone (IGF1Ri, 10 μM) decreased significantly the phosphorylation of P-IRS-1 (Ser794) and P-Akt (Thr308) (*p *< 0.0001, for P-IRS-1 expression in control *vs*. HNMPA(AM)_3 _treated cells; *p *< 0.001, for P-Akt expression in control *vs*. HNMPA(AM)_3 _treated cells), but had a negligible effect on P-Akt (Ser473). More important, co-treatment with an IGF-1R inhibitor in cells exposed to AICAR failed to restore the observed AICAR-induced up-regulation of P-IRS-1 (Ser794), and P-Akt (Thr308), while phosphorylation of Akt at Ser473 remained unaffected (Fig. 2, A + IGF1Ri). These findings indicate that AICAR-induced Akt phosphorylation at Thr308 is dependent of IGF-1R/IRS-1 activation whereas phosphorylation of Akt at Ser473 occurs independently of IGF-1R/IRS-1 signaling but requires AMPK activation. Therefore, AMPK activation by AICAR promotes activation of Akt by two mechanisms: phosphorylation of Akt (Thr308) by IGF-1R/IRS-1 (Ser794) signaling mediated by AMPK and its downstream down-regulation of mTOR, and the other through phosphorylation of Akt (Ser473) by an AMPK-dependent mechanism.

**Figure 3:**
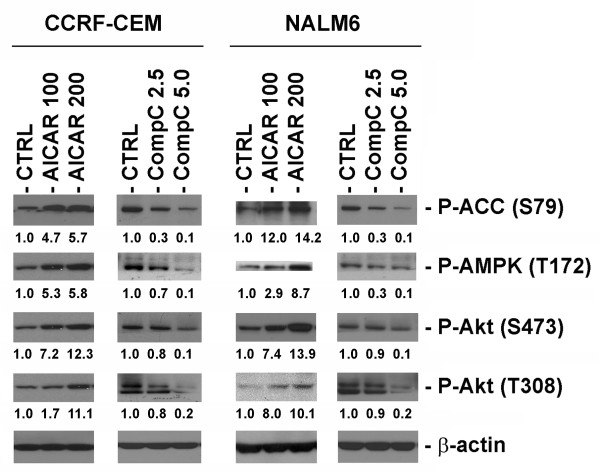
**Functional AMPK activity is required for activation of Akt at Ser473 and Thr308 in ALL cell lines**. CCRF-CEM and NALM6 cells were exposed to either the AMPK activator AICAR (100 and 200 μM) or the AMPK inhibitor compound-C (CompC, 2.5 and 5.0 μM) for 24 h at 37°C. Proteins were extracted and analyzed by Western immunoblotting for the expression of P-Akt (Ser473 and Thr308), P-AMPK (Thr172), and P-ACC (Ser79). The density value of each band was normalized to β-actin level and expressed relative to control (shown as fold induction).

To further investigate the role of AMPK in the activation of Akt, we compared the effects of the AMPK activator AICAR and compound-C, a known specific inhibitor of AMPK [32,33]. Western blot analysis of protein extracts from CCRF-CEM and NALM6 cells treated with either AICAR (100 & 200 μM) or compound-C (2.5 & 5.0 μM) showed that activation of AMPK correlated with phosphorylation of Akt at both residues (Ser473 and Thr308), and conversely inhibition of AMPK by compound-C also led to down-regulation P-Akt at both residues (Fig. 3). To ascertain the affects of P-AMPK in these experiments, the functional activation or inhibition of AMPK signaling were confirmed by the determining the phosphorylation status of P-ACC (Ser79). As seen in Fig. 3, expression of P-ACC directly correlated with the phosphorylation status of AMPK at Thr172. These data together with data presented in Fig. 2, strongly suggest that functional AMPK signaling is required for activation of Akt at both Ser473 and Thr308, but the phosphorylation of Akt at Thr308 also requires IGF-1R/IRS-1 signaling. Therefore, the compensatory activation of Akt seen in ALL cells following AICAR-induced AMPK activation resulted from phosphorylation of Akt at Thr308 and Ser473 (Fig. 2).

### Inhibition of IGF-1R tyrosine kinase activity with HNMPA(AM)_3 _induces growth inhibition and apoptosis in ALL cell lines

**Figure 4:**
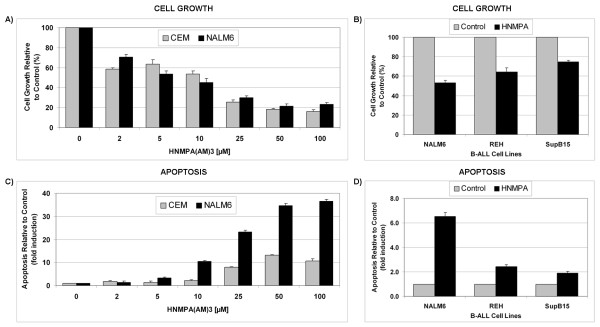
**Inhibition of IGF-1R tyrosine kinase activity induces growth inhibition and apoptosis in ALL cell lines**. Cell growth (**A**) and level of apoptosis (**C**) detected in ALL CCRF-CEM and NALM6 cells treated with the IGF-1R inhibitor HNMPA(AM)_3 _(2 - 100 μM) and incubated for 24 h at 37°C. Proliferation (**B**) and level of apoptosis (**D**) detected in Bp-ALL subtypes NALM6, REH (t[12;21]), and SupB15 (t[9;22]) treated with HNMPA(AM)_3 _(10 μM) and incubated for 24 h at 37°C. The cell growth (viability) values are expressed as a percentage relative to those obtained with untreated control cells (mean ± SEM, n = 3). Annexin V-FITC/PI staining data (apoptosis) were normalized and expressed as fold induction relative to control values (mean ± SEM, n = 3) as described in Methods.

Phosphorylation of Akt at Thr308 was shown to be sufficient to induce Akt's pro-survival effects [34] but phosphorylation of both residues is needed for optimal activity. To examine the role of IGF-1R/IRS-1 signaling in ALL cell survival, we evaluated the effects of IGF-1R inhibition using HNMPA(AM)_3 _(2 - 100 μM) on cell growth and apoptosis using a panel of ALL cell models. As shown in Fig. 4A, treatment of CCRF-CEM and NALM6 cells with HNMPA(AM)_3 _inhibited their growth in a dose-dependent manner with calculated EC50 values of 16.5 μM and 6.1 μM for CCRF-CEM and NALM6, respectively. We then extended our analysis to other Bp-ALL subtypes characterized by the non-random translocations REH [t(12;21)] and SupB15 [t(9;22)]. In NALM6 treatment with HNMPA(AM)_3 _(10 μM) led to 50% growth inhibition compared to 40% and 25% in REH and SupB15 cells, respectively (Fig. 4B).

To determine if IGF-1R inhibition was cytostatic or cytotoxic in ALL cells, we determined induction of apoptosis in these same cell models. CCRF-CEM and NALM6 cells were treated with increasing concentrations of HNMPA(AM)_3 _(2 - 100 μM) and apoptosis was assayed using Annexin V-FITC/PI staining. Fig. 4C shows that HNMPA(AM)_3 _induced apoptotic cell death in a dose-dependent manner in NALM6, and to a lower extent in CCRF-CEM cells. Comparatively, the maximal fold increase in apoptotic cell death was approximately 40-fold compared to control in NALM6 cells, whereas only a 10-fold increase in apoptotic death was observed in CCRF-CEM cells (Fig. 4C). Level of apoptosis in the Bp-ALL subtypes REH [t(12;21)] and SupB15 [t(9;22)] following treatment with HNMPA(AM)_3 _(10 μM) was significantly lower compared to NALM6 cells (*p *= 0.0032, for NALM6 *vs*. REH; *p *= 0.0016, for NALM6 *vs*. SupB15). REH and SupB15 cells exhibited only a 2-fold increase in apoptotic cell death compared to a 6-fold increase in NALM6 cells (Fig. 4D). Similar fold differences were observed over a range of drug concentrations. Interestingly, the translocation t(9;22) encoding for the BCR-ABL fusion protein expressed in SupB15 cells was shown to induce autocrine IGF-1 signaling in leukemia, which may confer clinical resistance due to higher IGF-1R signaling and constitutive P-Akt activity. Taken together, these data raise the intriguing possibility that cell lineage of origin (B- *vs*. T-ALL) and the presence of non-random translocations may modulate IGF-1R activity and consequently may influence ALL cell death *vs*. cell survival when exposed to IGF-1R inhibitors.

### Differential expression level of IGF-1R and downstream signaling factors in ALL cells

**Figure 5:**
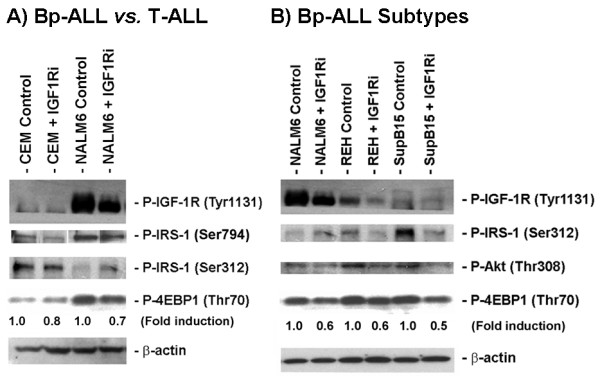
**Expression level of IGF-1R and downstream regulated signaling factors in B-ALL *vs*. T-ALL cell lines**. **A) **Western blot analysis of P-IGF-1R (Tyr1131), P-IRS-1 (Ser794 and Ser312), and P-4EBP1 (Thr70) expression in Bp-ALL (NALM6) and T-ALL cells (CCRF-CEM) treated with 0.1% DMSO (Control) or the IGF-1R inhibitor HNMPA(AM)_3 _(IGF1Ri, 10 μM) and incubated for 24 h. **B) **Basal expression level of P-IGF-1R (Tyr1131), P-IRS-1 (Ser312), P-Akt (Thr308), and P-4EBP1 (Thr70) in Bp-ALL NALM6 and subtypes characterized by non-random translocations (REH (t[12;21]), and SupB15 (t[9;22])). The levels of P-4EBP1 were normalized to β-actin (loading control) and expressed relative to control (shown as fold induction).

The different levels of sensitivity to the IGF-1R inhibitor observed among CCRF-CEM (T-ALL) and NALM6 (Bp-ALL) cells, and within Bp-ALL REH and SupB15 subtypes expressing selected non-random translocations prompted us to investigate the mechanism underlying these differences. To address this question, we performed Western blot analysis of key factors associated with the IGF-1R signaling cascade in these cell models. As shown in Fig. 5A, NALM6 cells expressed higher levels of phospho-IGF-1R (Tyr1131) and phospho-IRS-1 (Ser794) than CCRF-CEM cells. Similarly, lower levels of expression of P-IGF-1R (Tyr1131) and P-IRS-1 (Ser794) were detected in the Bp-ALL REH [t(12;21)] and SupB15 [t(9;22)] subtypes characterized by non-random translocations in comparison to NALM6 (Fig. 5B). In addition, the expression of P-Akt was higher in CCRF-CEM cells (shown in Fig. 3) and REH cells (Fig. 5B), which correlated with these cell models having either a mutation or a deletion in the PTEN gene, respectively [35,36]. Similarly, the high level of P-Akt found in SupB15 cells (carrying the BCR-ABL gene fusion) results from inhibition of PP1α, a serine phosphatase that negatively regulates the PI3K/Akt pathway [37]. Mechanistically, higher levels of IGF-1R/IRS-1 expression correlated with higher sensitivity to IGF-1R inhibition, with NALM6 cells exhibiting the highest expression of IGF-1R and the highest sensitivity to apoptotic cell death following IGF-1R inhibition. As expected, treatment with the IGF-1R inhibitor HNMPA(AM)_3 _(IGF1Ri, 10 μM) reduced considerably the expression of both IGF-1R and IRS-1 phosphorylated proteins (Fig. 5A). Additionally, phosphorylation of IRS-1 at Ser312, the residue targeted by mTOR and responsible for the negative feedback-loop inhibition, was inversely expressed compared to the expression of P-IGF-1R (Tyr1131) in all the cells examined (Fig. 5A and 5B). The activity of P-mTOR was monitored using P-4EBP1 (Thr70) expression, its immediate downstream target [38], and demonstrated that mTOR activity was down-regulated in all cell lines following IGF-1R inhibition. These data further suggest that "addiction" of the cells to IGF-1R activity as determined by P-IGF-1R (Tyr1131) and P-IRS-1 (Ser312) expression makes cells more dependent on IGF-1R signaling for survival, and therefore more susceptible to IGF-1R inhibition.

### Simultaneous inhibition of IGF-1R or Akt signaling pathways with AMPK activator induces synergistic cytotoxicity in ALL cell lines

**Figure 6:**
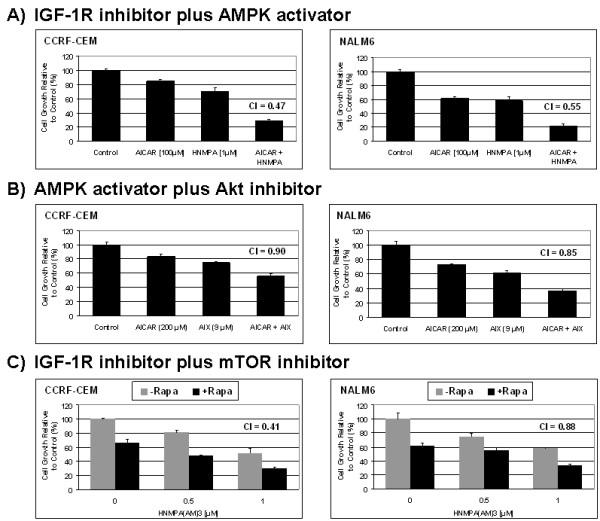
**Simultaneous inhibition of IGF-1R with drug altering AMPK, mTOR, or Akt signaling pathway induces synergistic growth inhibition in ALL cell lines**. Cell growth of ALL CCRF-CEM and NALM6 cells treated with either AICAR (100 μM) plus the IGF-1R inhibitor HNMPA(AM)_3 _(1.0 μM) (panel **A**); AICAR (200 μM) plus the Akt inhibitor-X (AIX, 9.0 μM) (panel **B**); or rapamycin (1.0 μg/ml) plus HNMPA(AM)_3 _(0.5 and 1.0 μM) (panel **C**). Cells were treated with either agent alone or in combination at the indicated doses and incubated for 24 h at 37°C. The cell growth values are expressed as a percentage relative to those obtained with control cells (mean ± SEM, n = 3). Combination index (CI) values were determined for each drug combination as described in Methods (CI <1 indicates synergism).

Our laboratory and others have demonstrated that significant functional cross-talk between AMPK, mTOR, IGF-1R/IRS-1, and Akt signaling factors occur in leukemia cells [3,9,39-42]. Since inhibition of IGF-1R activity is capable of inducing growth inhibition and apoptotic cell death, we reasoned that co-targeting these interconnected pathways would result in enhanced cytotoxicity. To test this hypothesis we tested three combination strategies in ALL cell line models. First, we evaluated agents targeting simultaneously the AMPK (AICAR, 100 μM) and IGF-1R (HNMPA(AM)_3_, 1 μM) signaling proteins. This combination resulted in significant growth inhibition (*p *< 0.001, for AICAR + HNMPA(AM)_3 _*vs*. control, AICAR alone, and HNMPA(AM)_3 _alone) in CCRF-CEM and NALM6 cell lines examined (Fig. 6A) with a calculated combination index (CI) of 0.47 and 0.55 for CCRF-CEM and NALM6, respectively. Second, we tested whether inhibition of Akt, downstream to IGF-1R signaling, in the presence of AICAR would also increase growth inhibition. As shown in Fig. 6B, combination of AICAR (200 μM) plus the Akt inhibitor X (AIX, 9 μM) had similar effects with CI values of 0.90 and 0.85 for CCRF-CEM (*p *< 0.001, for AICAR + AIX *vs*. control, AICAR alone, and AIX alone) and NALM6 (*p *< 0.05, for AICAR + AIX *vs*. control, AICAR alone, and AIX alone), respectively. These results suggest that blocking activation of Akt by either inhibiting IGF-1R/IRS-1 activity or the downstream interference with Akt phosphorylation, greatly increases the growth inhibition when AMPK is simultaneously activated by AICAR in ALL cells. Third, we tested the combined inhibition of IGF-1R and mTOR, which is negatively regulated following AMPK activation [3]. For these experiments we treated cells with the mTOR inhibitor rapamycin plus the IGF-1R inhibitor HNMPA(AM)_3_. Although blocking mTOR activity would have a negative effect on cell proliferation secondary to inhibition of protein synthesis, it would also relieve the feedback loop inhibition on IRS-1 activating Akt, which might promote cell growth. As presented in Fig. 6C, treatment of CCRF-CEM and NALM6 cells with rapamycin (1 μg/ml) and HNMPA(AM)_3 _(0.5 - 1.0 μM) induced growth inhibition with CI values of 0.41 and 0.88 for CCRF-CEM and NALM6 cells, respectively. Therefore, the three combination strategies tested resulted in synergistic growth inhibition in both cell lines examined, as evidenced by CI values <1 in all cases.

**Figure 7:**
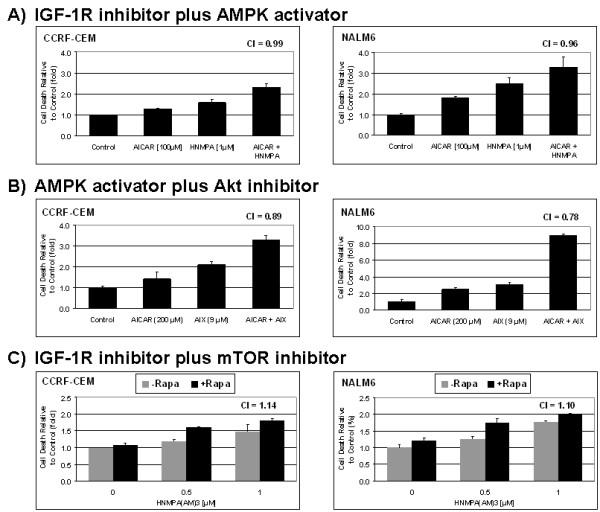
**Co-targeting AMPK and Akt signaling pathway induces synergistic cell death in ALL cell lines**. Cell death values obtained from the ALL CCRF-CEM and NALM6 cells shown in Fig. 6 which were treated with either AICAR (100 μM) plus the IGF-1R inhibitor HNMPA(AM)_3 _(1.0 μM) (panel **A**); AICAR (200 μM) plus the Akt inhibitor-X (AIX, 9.0 μM) (panel **B**); or rapamycin (1.0 μg/ml) plus HNMPA(AM)_3 _(0.5 and 1.0 μM) (panel **C**). The cell death values were generated from the trypan blue exclusion data and are expressed as a percentage relative to those obtained with control cells (mean ± SEM, n = 3). Combination index (CI) values were determined for each drug combination as described in Methods (C <1, = 1, and >1 indicate synergism, additive effect, and antagonism, respectively).

We then analyzed induction of the cell death resulting from these drug combinations and found that only the combination AICAR plus AIX, targeting AMPK and Akt simultaneously, was synergistic with a CI value of 0.89 and 0.78 for CCRF-CEM and NALM6 cell lines, respectively (Fig. 7B). Although additional cell death was observed for the other combinations as compared to single drug alone, none of the cytotoxic effects resulting from the two other drug combinations were synergistic (Fig. 7A and 7B). The combination HNMPA(AM)_3 _plus AICAR resulted in a "borderline" CI of 0.99 and was considered additive, whereas the combination of HNMPA(AM)3 plus rapamycin was found to be antagonistic with CI >1. In both cases in which combination therapy was either additive or synergistic in inducing cell death in NALM6 and CCRF-CEM cells, activation of AMPK signaling was co-targeted. These data suggest that the master energy regulator AMPK plays a pivotal role in triggering apoptotic cell death when these signaling cascades are co-targeted, and that the cross-talk between AMPK and the IGF-1R, Akt and mTOR pathways appears to be important in determining cellular fate following perturbations of these cascades. Taken together, our data indicate that blocking simultaneously both the key cell proliferation regulator mTOR, and the IGF-1R-induced Akt phosphorylation pathway resulted in significant cell growth inhibition and cell death by interfering with the mechanism of cell survival triggered by treatment with single agents.

## Discussion

In search for novel treatment strategies, we investigated AMPK signaling as potential target for ALL therapy. Our results, together with our previously published report [3] reveal that activation of AMPK by AICAR induces a compensatory survival response through activation of Akt at both of its functional residues Ser473 and Thr308. Although phosphorylation of Akt at both residues is critical for maximum catalytic activity [9,43], it has been established that phosphorylation of Thr308 is sufficient to activate its kinase activity and support cell survival [34]. We show that the mechanism of Akt activation in ALL cells is mediated in part by AMPK-induced phosphorylation of IRS-1 at Ser794, the immediate downstream effectors of the IGF-1R signaling cascade, and also in part by AMPK-induced inhibition of mTOR and its downstream feedback loop inhibition of IRS-1 (Ser312). Direct interaction between P-AMPK (Thr172) and phosphorylation of IRS-1 at Ser794 has been shown to occur in several systems such as cell lines [31,44,45], and insulin-resistant animal models [46], but the biological relevance of this phosphorylation event is still not clear. Different functions have been reported for AMPK-induced IGF-1R phosphorylation with some reporting a positive effect on PI3K/Akt signaling [31] whereas others reported a negative effect [45-47]. Additive activation of AMPK and Akt has been shown to regulate important biological functions such as angiogenesis and glucose metabolism [48,49], suggesting that positive interactions exist between AMPK and Akt as we report here. Other reports demonstrated that Akt could negatively regulate AMPK activity by direct binding and phosphorylation of AMPK at Ser485 [50-52]. These opposite effects reflect the complexity of the signaling cross-talk that exists between AMPK, IRS-1, and downstream activation of Akt.

It is clear from our studies that phosphorylation of Akt at Thr308 in AICAR-treated ALL cells occurs via direct AMPK down-regulation of mTOR and activation of the IGF-1R/IRS-1 signaling cascade. This compensatory mechanism promotes cell survival because inhibition of IGF-1R activity in either presence or absence of AICAR decreases P-IRS-1 (Ser794) and P-Akt (Thr308) levels and significantly increases apoptotic cell death. The signaling cascade triggered by activation of tyrosine kinase receptor leading to phosphorylation of IRS-1 and subsequent activation Akt at Thr308 have been extensively studied and is mediated by the downstream PI3K and PDK1 kinases [16,34,53]. Additionally, we demonstrate that phosphorylation of Akt is also dependent on AMPK since inhibition of AMPK activity with compound-C clearly decreased P-Akt at both residues. AMPK has been shown to inhibit mTORC1 activity by two different mechanisms: one through activation of the TSC2, which promotes downstream inhibition of the mTOR activator Rheb [9,54], and the other through direct phosphorylation of Raptor at Ser792 blocking mTORC1 activation [55]. Additional studies demonstrated that phosphorylation of Akt at Ser473 was mediated by mTORC2, a complex formed by the association of rictor, mSin1, mLST8, with mTOR [7,10,12,34,56,57]. Among mTORC1 and mTORC2, mTOR is the only critical factor that is shared by both complexes [9]. Thus, it is tempting to speculate that by down-regulating mTORC1, AMPK could increase the availability of mTOR and favor the formation of mTORC2, which would promote phosphorylation of Akt at Ser473. A similar mechanism was proposed for activation of Akt by AMPK in macrophages expressing a constitutively active form of AMPK [58]. Nevertheless, we can not rule out the possibility that a distinct mechanism independent of mTORC2 might be involved in this process.

The data presented herein shows that activity of IGF-1R/IRS-1 was higher in NALM6 *vs*. CCRF-CEM cells, and that their expression also differs within Bp-ALL REH and SupB15 subtypes characterized by the non-random translocations t[12;21], and t[9;22]. More important, these differences correlated with reduction in P-IRS-1 (Ser794) and P-Akt (Ser473 and Thr308), and degree of induction of apoptotic death resulting from the pharmacological inhibition of IGF-1R. Our results raise the intriguing possibility that cell lineage of origin and/or presence of selected non-random translocations may influence response to therapy in ALL cells treated with inhibitors of IGF-1R. This possibility needs to be investigated using primary samples from patients with ALL. It is also possible that the level of Akt activation in these cells may also dictate their degree of sensitivity to IGF-1R inhibition. For instance, it is well known that the CCRF-CEM cell line carries a mutation inactivating PTEN [35] and that REH cells born a PTEN deletion [36], both leading to increased reliance on Akt signaling for cell survival. In addition, SupB15 cells express high levels of P-Akt because the expression of the BCR-ABL gene fusion inhibits PP1α, a serine phosphatase that negatively regulates the PI3K/Akt pathway [37]. Interestingly, the expression level of P-Akt was the lowest in NALM6 cells which was also the most sensitive to the IGF-1R inhibitor HNMPA(AM)_3 _as compared to all of the other cell lines examined, therefore suggesting that Akt provides a mechanism to escape cell death following IGF-1R inhibition.

To further assess whether IGF-1R signaling may be influenced by biological pathways closely linked to cell lineage and non-random chromosomal translocations, we have mined existing gene expression databases from childhood ALL patients http://www.stjuderesearch.org/data/ALL1, and found that the expression of relevant IGF-1 regulatory carriers such as IGFBP2 and IGFBP4 appear to be significantly differentially expressed in ALL in a phenotype specific manner. The known correlation between these carriers and IGF-1 suggested to us that differences in IGF-1 signaling may exist in ALL, and impact critical oncogenic and survival signaling pathways. Interestingly, IGF-1R signaling has been linked to cell lineage of origin in ALL. For instance, significant differences in the expression of the IGF-1 system components IGF-II, IGFBP-2, IGFBP-4 and IGFBP-5 have been described between B-lineage and T-lineage ALL [28-30]. IGFBP-2 was identified as the major regulatory carrier in childhood leukemia and exhibited an inverse correlation with IGF-1 levels [59], suggesting that activation of IGF-1R signaling may confer ALL cells a survival advantage and influence induction of apoptosis. Emerging literature suggests that IGF-1R signaling may also be influenced by non-random translocations in ALL [60,61]. For instance, leukemia cells expressing the translocation t(9;22) encoding for the BCR-ABL fusion not only exhibit a higher degree of resistance to chemotherapeutic drugs but also were shown to induce autocrine IGF-1 signaling. Thus, it is clear that IGF-1R pathway may provide ALL cells a survival advantage through its crosstalk with other critical metabolic networks.

The identification of potential cross-talk within compensatory survival pathways in ALL cells prompted us to developed simultaneous co-targeting strategies to induce cell death in ALL cells. We demonstrated that co-targeting IGF-1R and downstream pathways (AMPK & IGF-1R, mTOR & IGF-1R, and AMPK & Akt) led to synergistic growth inhibition in ALL cell models. This is consistent with the study of Bertrand *et al*. [62] that demonstrated that blocking IGF-1R activity using an antibody synergized with inhibitors of PI3K/Akt/mTOR pathway by suppressing the IGF-1R-induced Akt phosphorylation, and consequently promoted apoptosis in hematopoietic cells. Among the three drug combinations tested, only the one co-targeting AMPK and Akt resulted in synergistic induction of cell death. This can be explained in part by differences in the mechanism of action between AIX *vs*. HNMPA(AM)_3_, with AIX being more effective in inactivating Akt. Taken together, rationally designed simultaneous targeting of key factors within the AMPK, IGF-1R, and mTOR pathways leads to synergistic induction of cell growth inhibition by blocking compensatory survival responses triggered by treatment with single agents. Nevertheless, of the combinations strategies tested only co-targeting AMPK plus Akt lead to synergistic induction of apoptosis.

## Conclusions

We conclude that IGF-1R and its downstream metabolic and oncogenic pathways contribute to cell survival and are important to determine pro- or anti-apoptotic responses in ALL cells to treatment with inhibitors of these signaling pathways. Our data suggest that PTEN status, AMPK and Akt signaling, and possibly cell-lineage and non-random translocations, influence IGF-1R signaling and sensitivity to IGF-1R inhibitors in ALL lymphoblasts. Selected combination strategies aimed at inhibiting IGF-1R and related downstream pathways represent a potential strategy for future translation into novel ALL therapies, in particular when AMPK is one of the signaling proteins targeted in these combinations.

## Methods

### Cell lines and chemicals

The childhood ALL cell lines CCRF-CEM (T-lineage), NALM6 (Bp-lineage), REH (Bp-ALL/t(12;21)) were grown in RPMI 1640 medium supplemented with 10% FBS and antibiotics. SupB15 (Bp-ALL/t(9;22)) were grown in Iscove's modified DMEM medium with 20% FBS. Cell culture media were purchased from Cellgro (Mediatech, Inc, Manassas, VA). All other cell culture reagents were from Invitrogen Corporation (Carlsbad, CA). Cells were treated with agents known to activate AMPK (AICAR, Toronto Research Chemicals, Inc, Ontario, Canada)) or inhibit IGF-1R (HNMPA(AM)_3_), mTOR (rapamycin, LC Laboratories, Woburn, MA), or Akt (Akt inhibitor-X, EMD Chemicals, Inc., Gibbstown, NJ), and incubated for periods of 24 to 48 h (5% CO2 at 37°C).

### Cell proliferation assays

Cell viability (500 μl) was determined using the Vi Cell XR system (Beckman-Coulter), and values are expressed as a percentage relative to those obtained in untreated controls (means ± SEM, n = 3). Synergism was determined using the Chou's combination index (CI) based on the following equation: CI = [(D_1 combination_/D_1 single_) + (D_2 combination_/D_2 single_)] [63]. The numerators D_1 combination _and D_2 combination _represent the concentration of the drug D_1 _and D_2, _respectively, used in the combination treatment that inhibits cell growth by x%. The denominators D_1 single _and D_2 single _represent the concentration of drug D1 and D2 as single agent needed to achieve the same level of growth inhibition than in the combination (x%).

### Apoptosis assays

Apoptosis was evaluated using the Annexin V-FITC Apoptosis Detection Kit I following the manufacturer's recommendations (BD Biosciences, San Jose, CA). Briefly, cells were washed twice with 1× PBS pH 7.4, resuspended to [1 × 10^6 ^cells/ml] in 1× Binding Buffer, then 100 μl of cells were incubated with a mixture of Annexin V/Propidium Iodide (PI) reagents for 15 min/RT°C, equilibrated with 400 μl 1× Binding Buffer, and fluorescence was analyzed by flow cytometry (BD Biosciences LSR II flow cytometer; University of Miami-SCCC Flow Cytometry Core Facility). Apoptotic Annexin V/PI staining values were combined, and normalized to control values (fold induction).

### Western immunoblotting

Protein extracts were prepared by sonication in the presence of protease inhibitors, and quantified using the Micro BCA Protein Assay Kit (Pierce, Rockford, IL). Proteins (50 μg/lane) were resolved by 4-15% SDS-PAGE (Bio-Rad, Hercules, CA), transferred onto PVDF membranes (Invitrogen, Carlsbad, CA) and immunodetected using a Western Lighting ECL system (Perkin Elmer, Waltham, MA). For immunodetection of P-AMPK (Thr172), P-Akt (Ser473 & Thr308), P-IRS-1 (Ser312 & Ser794), P-IGF-1R (Tyr1131), P-4EBP1 (Thr70), P-mTOR (Ser2448), and β-actin, we used specific primary antibody against each protein and horseradish peroxidase-conjugated secondary antibody (anti IgG) (Cell Signaling, Danvers, MA). Expression of each protein was determined by densitometry analysis of the immunodetected bands, normalized to β-actin, and expressed relative to control (fold induction). The immunoblots shown are representative of 3 independent experiments, which produced similar results.

## Abbreviations list

ACC: acetyl-CoA carboxylase; AICAR: 5-aminoimidazole-4-carboxamide 1-D-ribonucleoside; AIX: Akt inhibitor X; ALL: acute lymphoblastic leukemia; Akt: protein kinase B; AMPK: AMP activated protein kinase; HNMPA(AM)_3_: hydroxy-2-naphthalenylmethylphosphonic acid trisacetoxymethyl ester; IGF-1R: insulin-like growth factor-1 receptor; IGFBP: IGF-binding protein; IRS-1: insulin receptor substrate-1; PDK1: phosphoinositide-dependent kinase-1; PI3K: phosphoinositide 3-kinase; P1P3: phosphatidylinositol (3,4,5)-triphosphate; PTEN: phosphatase and tensin homolog; mTOR: mammalian target of rapamycin; 4EBP1: eukaryotic translation initiation factor 4E binding protein-1; TORC: mTOR complex; MAPK: mitogen activated protein kinase.

## Competing interests

The authors declare that they have no competing interests.

## Authors' contributions

GML carried out major experiments including Western blots, cell growth proliferation and apoptosis assays, statistical data analysis, conceived the study and participated in designing the experiments. GJL participated in data analysis, flow-cytometry assays, and in designing the experiments. GF carried out Western blots. JCB is the corresponding author and is responsible for experimental design and coordination. All authors read and approved the final manuscript.

## References

[B1] Pui CH Evans WE Treatment of acute lymphoblastic leukemia N Engl J Med 2006 354 166 178 10.1056/NEJMra052603 16407512

[B2] Yeoh EJ Ross ME Shurtleff SA Williams WK Patel D Mahfouz R Classification, subtype discovery, and prediction of outcome in pediatric acute lymphoblastic leukemia by gene expression profiling Cancer Cell 2002 1 133 143 10.1016/S1535-6108(02)00032-6 12086872

[B3] Sengupta TK Leclerc GM Hsieh Kinser TT Leclerc GJ Singh I Barredo JC Cytotoxic effect of 5-aminoimidazole-4-carboxamide-1-beta-4-ribofuranoside (AICAR) on childhood acute lymphoblastic leukemia (ALL) cells: implication for targeted therapy Mol Cancer 2007 6 46 10.1186/1476-4598-6-46 17623090 PMC1948012

[B4] Datta SR Dudek H Tao X Masters S Fu H Gotoh Y Akt phosphorylation of BAD couples survival signals to the cell-intrinsic death machinery Cell 1997 91 231 241 10.1016/S0092-8674(00)80405-5 9346240

[B5] del Peso L Gonzalez-Garcia M Page C Herrera R Nunez G Interleukin-3-induced phosphorylation of BAD through the protein kinase Akt Science 1997 278 687 689 10.1126/science.278.5338.687 9381178

[B6] Virdee K Parone PA Tolkovsky AM Phosphorylation of the pro-apoptotic protein BAD on serine 155, a novel site, contributes to cell survival Curr Biol 2000 10 1151 1154 10.1016/S0960-9822(00)00702-8 10996800

[B7] Sarbassov DD Guertin DA Ali SM Sabatini DM Phosphorylation and regulation of Akt/PKB by the rictor-mTOR complex Science 2005 307 1098 1101 10.1126/science.1106148 15718470

[B8] Alessi DR Andjelkovic M Caudwell B Cron P Morrice N Cohen P Mechanism of activation of protein kinase B by insulin and IGF-1 Embo J 1996 15 6541 6551 8978681 PMC452479

[B9] Bhaskar PT Hay N The two TORCs and Akt Dev Cell 2007 12 487 502 10.1016/j.devcel.2007.03.020 17419990

[B10] Hresko RC Mueckler M mTOR.RICTOR is the Ser473 kinase for Akt/protein kinase B in 3T3-L1 adipocytes J Biol Chem 2005 280 40406 40416 10.1074/jbc.M508361200 16221682

[B11] Bayascas JR Leslie NR Parsons R Fleming S Alessi DR Hypomorphic mutation of PDK1 suppresses tumorigenesis in PTEN(+/-) mice Curr Biol 2005 15 1839 1846 10.1016/j.cub.2005.08.066 16243031

[B12] Breuleux M Klopfenstein M Stephan C Doughty CA Barys L Maira SM Increased AKT S473 phosphorylation after mTORC1 inhibition is rictor dependent and does not predict tumor cell response to PI3K/mTOR inhibition Mol Cancer Ther 2009 8 742 753 10.1158/1535-7163.MCT-08-0668 19372546 PMC3440776

[B13] Sale EM Sale GJ Protein kinase B: signalling roles and therapeutic targeting Cell Mol Life Sci 2008 65 113 127 10.1007/s00018-007-7274-9 17952368 PMC11131913

[B14] Martelli AM Nyakern M Tabellini G Bortul R Tazzari PL Evangelisti C Phosphoinositide 3-kinase/Akt signaling pathway and its therapeutical implications for human acute myeloid leukemia Leukemia 2006 20 911 928 10.1038/sj.leu.2404245 16642045

[B15] Shi Y Yan H Frost P Gera J Lichtenstein A Mammalian target of rapamycin inhibitors activate the AKT kinase in multiple myeloma cells by up-regulating the insulin-like growth factor receptor/insulin receptor substrate-1/phosphatidylinositol 3-kinase cascade Mol Cancer Ther 2005 4 1533 1540 10.1158/1535-7163.MCT-05-0068 16227402

[B16] Shah OJ Wang Z Hunter T Inappropriate activation of the TSC/Rheb/mTOR/S6K cassette induces IRS1/2 depletion, insulin resistance, and cell survival deficiencies Curr Biol 2004 14 1650 1656 10.1016/j.cub.2004.08.026 15380067

[B17] Aguirre V Uchida T Yenush L Davis R White MF The c-Jun NH(2)-terminal kinase promotes insulin resistance during association with insulin receptor substrate-1 and phosphorylation of Ser(307) J Biol Chem 2000 275 9047 9054 10.1074/jbc.275.12.9047 10722755

[B18] Schmitz-Peiffer C Whitehead JP IRS-1 regulation in health and disease IUBMB Life 2003 55 367 374 10.1080/1521654031000138569 14584587

[B19] Wan X Harkavy B Shen N Grohar P Helman LJ Rapamycin induces feedback activation of Akt signaling through an IGF-1R-dependent mechanism Oncogene 2007 26 1932 1940 10.1038/sj.onc.1209990 17001314

[B20] Baserga R Peruzzi F Reiss K The IGF-1 receptor in cancer biology Int J Cancer 2003 107 873 877 10.1002/ijc.11487 14601044

[B21] De Meyts P Whittaker J Structural biology of insulin and IGF1 receptors: implications for drug design Nat Rev Drug Discov 2002 1 769 783 10.1038/nrd917 12360255

[B22] Jones JI Clemmons DR Insulin-like growth factors and their binding proteins: biological actions Endocr Rev 1995 16 3 34 7758431 10.1210/edrv-16-1-3

[B23] Nakae J Kido Y Accili D Distinct and overlapping functions of insulin and IGF-I receptors Endocr Rev 2001 22 818 835 10.1210/er.22.6.818 11739335

[B24] Ryan PD Goss PE The emerging role of the insulin-like growth factor pathway as a therapeutic target in cancer Oncologist 2008 13 16 24 10.1634/theoncologist.2007-0199 18245009

[B25] Renehan AG Zwahlen M Minder C O'Dwyer ST Shalet SM Egger M Insulin-like growth factor (IGF)-I, IGF binding protein-3, and cancer risk: systematic review and meta-regression analysis Lancet 2004 363 1346 1353 10.1016/S0140-6736(04)16044-3 15110491

[B26] Ross JA Perentesis JP Robison LL Davies SM Big babies and infant leukemia: a role for insulin-like growth factor-1? Cancer Causes Control 1996 7 553 559 10.1007/BF00051889 8877054

[B27] Merchav S The haematopoietic effects of growth hormone and insulin-like growth factor-I J Pediatr Endocrinol Metab 1998 11 677 685 9829220 10.1515/jpem.1998.11.6.677

[B28] Vorwerk P Wex H Hohmann B Mohnike K Schmidt U Mittler U Expression of components of the IGF signalling system in childhood acute lymphoblastic leukaemia Mol Pathol 2002 55 40 45 10.1136/mp.55.1.40 11836446 PMC1187145

[B29] Wex H Ahrens D Hohmann B Redlich A Mittler U Vorwerk P Insulin-like growth factor-binding protein 4 in children with acute lymphoblastic leukemia Int J Hematol 2005 82 137 142 10.1532/IJH97.E0429 16146846

[B30] Mohnike KL Kluba U Mittler U Aumann V Vorwerk P Blum WF Serum levels of insulin-like growth factor-I, -II and insulin-like growth factor binding proteins -2 and -3 in children with acute lymphoblastic leukaemia Eur J Pediatr 1996 155 81 86 10.1007/BF02075755 8775218

[B31] Jakobsen SN Hardie DG Morrice N Tornqvist HE 5'-AMP-activated protein kinase phosphorylates IRS-1 on Ser-789 in mouse C2C12 myotubes in response to 5-aminoimidazole-4-carboxamide riboside J Biol Chem 2001 276 46912 46916 10.1074/jbc.C100483200 11598104

[B32] McCullough LD Zeng Z Li H Landree LE McFadden J Ronnett GV Pharmacological inhibition of AMP-activated protein kinase provides neuroprotection in stroke J Biol Chem 2005 280 20493 20502 10.1074/jbc.M409985200 15772080

[B33] Zhou G Myers R Li Y Chen Y Shen X Fenyk-Melody J Role of AMP-activated protein kinase in mechanism of metformin action J Clin Invest 2001 108 1167 1174 11602624 10.1172/JCI13505 PMC209533

[B34] Jacinto E Facchinetti V Liu D Soto N Wei S Jung SY SIN1/MIP1 maintains rictor-mTOR complex integrity and regulates Akt phosphorylation and substrate specificity Cell 2006 127 125 137 10.1016/j.cell.2006.08.033 16962653

[B35] Sakai A Thieblemont C Wellmann A Jaffe ES Raffeld M PTEN gene alterations in lymphoid neoplasms Blood 1998 92 3410 3415 9787181

[B36] Dahia PL Aguiar RC Alberta J Kum JB Caron S Sill H PTEN is inversely correlated with the cell survival factor Akt/PKB and is inactivated via multiple mechanismsin haematological malignancies Hum Mol Genet 1999 8 185 193 10.1093/hmg/8.2.185 9931326

[B37] Naughton R Quiney C Turner SD Cotter TG Bcr-Abl-mediated redox regulation of the PI3K/AKT pathway Leukemia 2009 23 1432 1440 10.1038/leu.2009.49 19295548

[B38] Gingras AC Raught B Sonenberg N mTOR signaling to translation Curr Top Microbiol Immunol 2004 279 169 197 14560958 10.1007/978-3-642-18930-2_11

[B39] Wullschleger S Loewith R Hall MN TOR signaling in growth and metabolism Cell 2006 124 471 484 10.1016/j.cell.2006.01.016 16469695

[B40] Tamburini J Chapuis N Bardet V Park S Sujobert P Willems L Mammalian target of rapamycin (mTOR) inhibition activates phosphatidylinositol 3-kinase/Akt by up-regulating insulin-like growth factor-1 receptor signaling in acute myeloid leukemia: rationale for therapeutic inhibition of both pathways Blood 2008 111 379 382 10.1182/blood-2007-03-080796 17878402

[B41] Manning BD Cantley LC AKT/PKB signaling: navigating downstream Cell 2007 129 1261 1274 10.1016/j.cell.2007.06.009 17604717 PMC2756685

[B42] Martelli AM Evangelisti C Chiarini F McCubrey JA The phosphatidylinositol 3-kinase/Akt/mTOR signaling network as a therapeutic target in acute myelogenous leukemia patients Oncotarget 2010 1 89 103 20671809 10.18632/oncotarget.114 PMC2911128

[B43] Scheid MP Marignani PA Woodgett JR Multiple phosphoinositide 3-kinase-dependent steps in activation of protein kinase B Mol Cell Biol 2002 22 6247 6260 10.1128/MCB.22.17.6247-6260.2002 12167717 PMC134003

[B44] Horike N Takemori H Katoh Y Doi J Min L Asano T Adipose-specific expression, phosphorylation of Ser794 in insulin receptor substrate-1, and activation in diabetic animals of salt-inducible kinase-2 J Biol Chem 2003 278 18440 18447 10.1074/jbc.M211770200 12624099

[B45] Tzatsos A Tsichlis PN Energy depletion inhibits phosphatidylinositol 3-kinase/Akt signaling and induces apoptosis via AMP-activated protein kinase-dependent phosphorylation of IRS-1 at Ser-794 J Biol Chem 2007 282 18069 18082 10.1074/jbc.M610101200 17459875

[B46] Qiao LY Zhande R Jetton TL Zhou G Sun XJ In vivo phosphorylation of insulin receptor substrate 1 at serine 789 by a novel serine kinase in insulin-resistant rodents J Biol Chem 2002 277 26530 26539 10.1074/jbc.M201494200 12006586

[B47] Ning J Clemmons DR AMP-activated protein kinase inhibits IGF-I signaling and protein synthesis in vascular smooth muscle cells via stimulation of insulin receptor substrate 1 S794 and tuberous sclerosis 2 S1345 phosphorylation Mol Endocrinol 2010 24 1218 1229 10.1210/me.2009-0474 20363874 PMC2875806

[B48] Ouchi N Kobayashi H Kihara S Kumada M Sato K Inoue T Adiponectin stimulates angiogenesis by promoting cross-talk between AMP-activated protein kinase and Akt signaling in endothelial cells J Biol Chem 2004 279 1304 1309 10.1074/jbc.M310389200 14557259 PMC4374490

[B49] Bertrand L Ginion A Beauloye C Hebert AD Guigas B Hue L AMPK activation restores the stimulation of glucose uptake in an in vitro model of insulin-resistant cardiomyocytes via the activation of protein kinase B Am J Physiol Heart Circ Physiol 2006 291 H239 250 10.1152/ajpheart.01269.2005 16489105

[B50] Berggreen C Gormand A Omar B Degerman E Goransson O Protein kinase B activity is required for the effects of insulin on lipid metabolism in adipocytes Am J Physiol Endocrinol Metab 2009 296 E635 646 10.1152/ajpendo.90596.2008 19158325

[B51] Horman S Vertommen D Heath R Neumann D Mouton V Woods A Insulin antagonizes ischemia-induced Thr172 phosphorylation of AMP-activated protein kinase alpha-subunits in heart via hierarchical phosphorylation of Ser485/491 J Biol Chem 2006 281 5335 5340 10.1074/jbc.M506850200 16340011

[B52] Kovacic S Soltys CL Barr AJ Shiojima I Walsh K Dyck JR Akt activity negatively regulates phosphorylation of AMP-activated protein kinase in the heart J Biol Chem 2003 278 39422 39427 10.1074/jbc.M305371200 12890675

[B53] Haruta T Uno T Kawahara J Takano A Egawa K Sharma PM A rapamycin-sensitive pathway down-regulates insulin signaling via phosphorylation and proteasomal degradation of insulin receptor substrate-1 Mol Endocrinol 2000 14 783 794 10.1210/me.14.6.783 10847581

[B54] Inoki K Zhu T Guan KL TSC2 mediates cellular energy response to control cell growth and survival Cell 2003 115 577 590 10.1016/S0092-8674(03)00929-2 14651849

[B55] Gwinn DM Shackelford DB Egan DF Mihaylova MM Mery A Vasquez DS AMPK phosphorylation of raptor mediates a metabolic checkpoint Mol Cell 2008 30 214 226 10.1016/j.molcel.2008.03.003 18439900 PMC2674027

[B56] Shiota C Woo JT Lindner J Shelton KD Magnuson MA Multiallelic disruption of the rictor gene in mice reveals that mTOR complex 2 is essential for fetal growth and viability Dev Cell 2006 11 583 589 10.1016/j.devcel.2006.08.013 16962829

[B57] Guertin DA Stevens DM Thoreen CC Burds AA Kalaany NY Moffat J Ablation in mice of the mTORC components raptor, rictor, or mLST8 reveals that mTORC2 is required for signaling to Akt-FOXO and PKCalpha, but not S6K1 Dev Cell 2006 11 859 871 10.1016/j.devcel.2006.10.007 17141160

[B58] Sag D Carling D Stout RD Suttles J Adenosine 5'-monophosphate-activated protein kinase promotes macrophage polarization to an anti-inflammatory functional phenotype J Immunol 2008 181 8633 8641 19050283 10.4049/jimmunol.181.12.8633 PMC2756051

[B59] Vorwerk P Mohnike K Wex H Rohl FW Zimmermann M Blum WF Insulin-like growth factor binding protein-2 at diagnosis of childhood acute lymphoblastic leukemia and the prediction of relapse risk J Clin Endocrinol Metab 2005 90 3022 3027 10.1210/jc.2004-0461 15687344

[B60] Grier DG Thompson A Kwasniewska A McGonigle GJ Halliday HL Lappin TR The pathophysiology of HOX genes and their role in cancer J Pathol 2005 205 154 171 10.1002/path.1710 15643670

[B61] Whelan JT Ludwig DL Bertrand FE HoxA9 induces insulin-like growth factor-1 receptor expression in B-lineage acute lymphoblastic leukemia Leukemia 2008 22 1161 1169 10.1038/leu.2008.57 18337761

[B62] Bertrand FE Steelman LS Chappell WH Abrams SL Shelton JG White ER Synergy between an IGF-1R antibody and Raf/MEK/ERK and PI3K/Akt/mTOR pathway inhibitors in suppressing IGF-1R-mediated growth in hematopoietic cells Leukemia 2006 20 1254 1260 10.1038/sj.leu.2404217 16642049

[B63] Chou TC Theoretical basis, experimental design, and computerized simulation of synergism and antagonism in drug combination studies Pharmacol Rev 2006 58 621 681 10.1124/pr.58.3.10 16968952

